# Supplementary material for: Nanocompartment-confined polymerization in living systems
Source: Nat Commun. 2023 Aug 26;14:5229. doi: 10.1038/s41467-023-40935-1 (PMC10460442; doi:10.1038/s41467-023-40935-1)
Supplement: Supplementary file 1 — Supplementary Information [file 41467_2023_40935_MOESM1_ESM.pdf]

Supplementary Information

Nanocompartment-Confined Polymerization in Living Systems

Chen et al.

Additional Experimental Details

Characterization: The transmission electron microscopy (TEM) images were collected by a JEOL JEM 2100plus microscopy at an accelerating voltage of 200 kV. The fluorescence spectrum was obtained on an RF5301 PC fluorescence spectrometer. Ultraviolet/visible/near–infrared absorption spectra (UV–vis/NIR) were measured on a Shimadzu UV-3600 spectrometer. Confocal laser scanning microscopy images were acquired by ZEISS LSM 800 microscopy. Flow cytometry was performed with Fortessa X20 (BD Biosciences). The photoacoustic signal was measured by LOIS-3D (TomoWave Laboratories, USA).

Chemicals: p-Aminodiphenylamine (ADPA), aniline (ANI), bromelain (Bro), horseradish peroxidase (HRP), 4,4'-iminodianiline (NDA), Irgacure 2959, m-phenylenediamine (MPD), o-phenylenediamine (OPD), p-phenylenediamine (PPD), sodium bis(2-ethylhexyl) sulfosuccinate (AOT), and sodium salt of 4-styrenesulfonate (NaSS) were purchased from Sigma-Aldrich and used without further purification. Anti-calreticulin antibody (cat no. ab227444, dilution of 1:200), anti-HMGB1 antibody (cat no. ab79823, dilution of 1:250), alexa fluor 488 conjugated goat anti-rabbit IgG H&L (cat no. ab150077, dilution of 1:1000), and goat anti-rabbit IgG H&L (cat no. Alexa Fluor® 594) (cat no. ab150080, dilution of 1:1000) were purchased from Abcam Inc. (Cambridge, CA, USA). Alexa Fluor®700 anti-mouse CD45 (cat no. 103128, dilution of 1:200), APC anti-mouse CD8a (cat no. 100712, dilution of 1:80), APC anti-mouse CD11c (cat no. 117310, dilution of 1:80), FITC anti-mouse CD3 (cat no. 100204, dilution of 1:50), FITC anti-mouse CD80 (cat no. 104706, dilution of 1:50), PE anti-mouse CD4 (cat no. 130310, dilution of 1:80), PE anti-mouse CD86 (cat no. 105008, dilution of 1:200), and purified anti-mouse CD16/32 (cat no. 156604, dilution of 1:200) were purchased from Biolegend. Secondary antibody CY3-goat anti-rabbit IgG (cat no. GB21303, dilution of 1:300) was purchased from Servicebio.

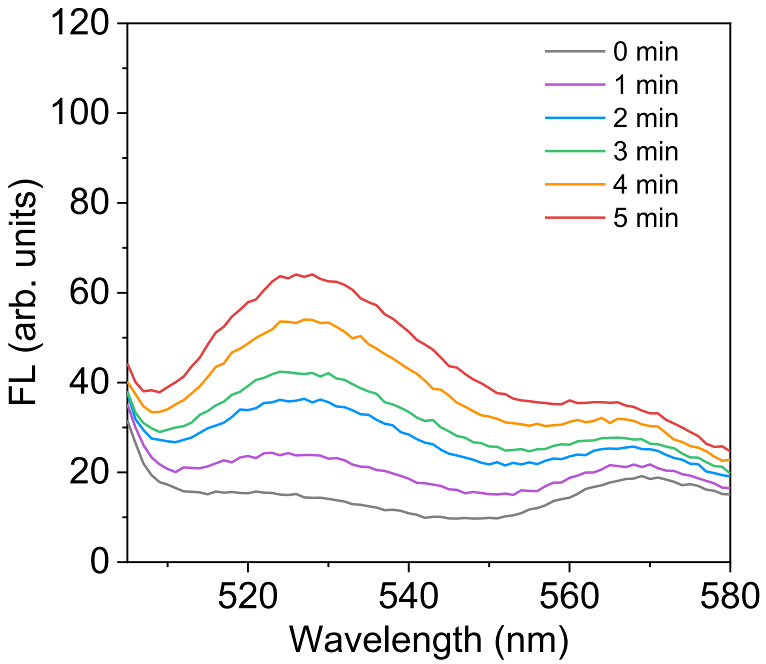

Supplementary Figure 1. Emission spectrum (λ_{ex} = 480 nm) of NaSS before and after UV illumination (365 nm).

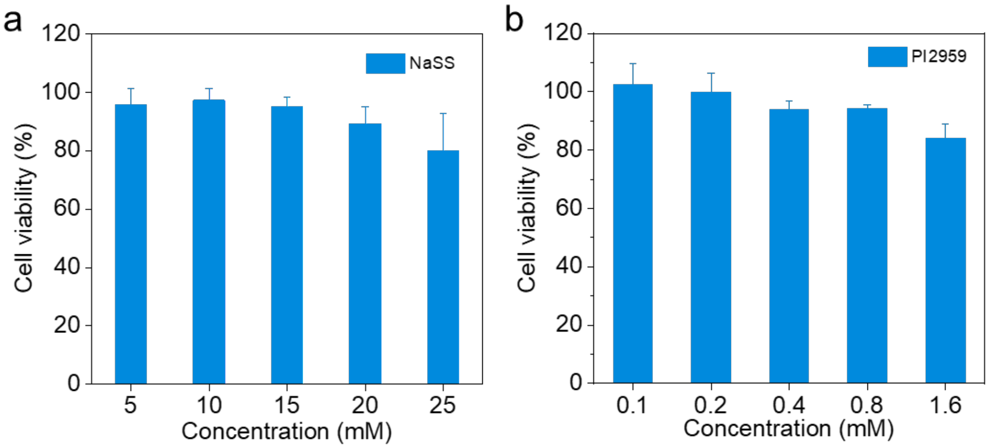

Supplementary Figure 2. Cell viability. Cell viability of 4T1 cells after incubation with **a** NaSS and **b** Irgacure 2959 (n = 5).

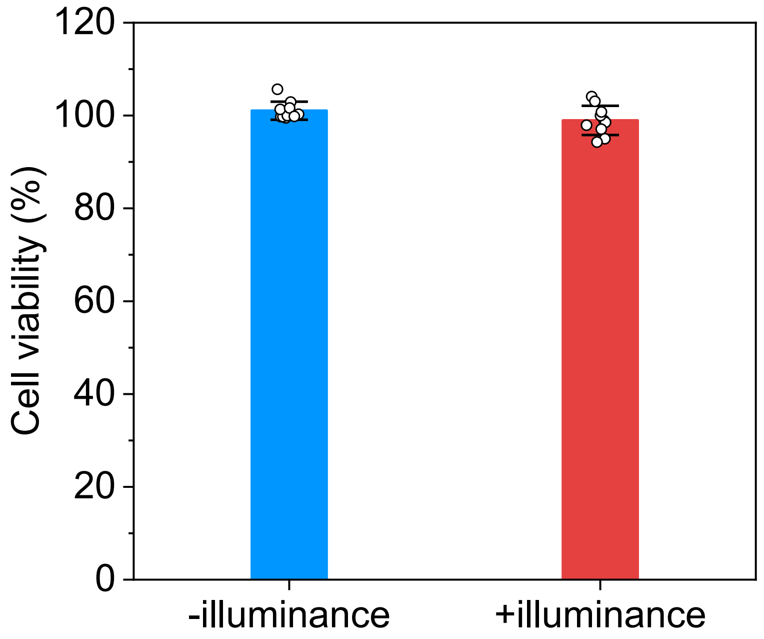

Supplementary Figure 3. Cell viability of 4T1 cells with or without illumination (365 nm, 5 min, n = 10).

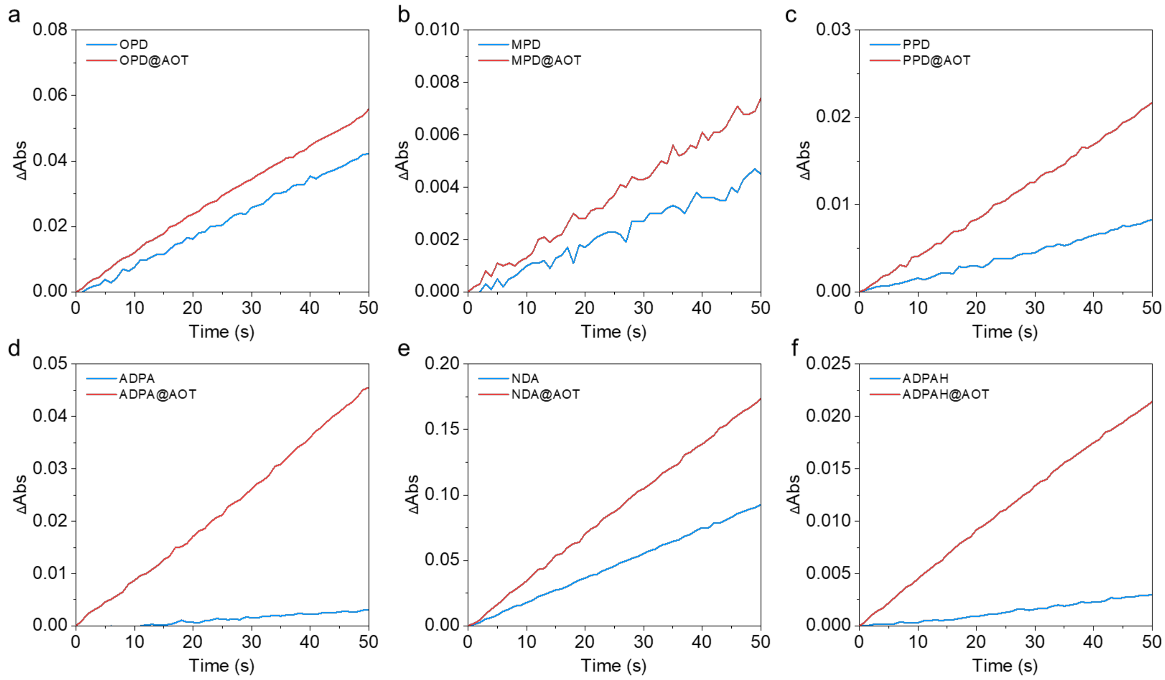

Supplementary Figure 4. Reaction rates. Reaction rates of **a** OPD and OPD@AOT, **b** MPD and MPD@AOT, **c** PPD and PPD@AOT, **d** ADPA and ADPA@AOT, **e** NDA and NDA@AOT, and **f** ADPAH and ADPAH@AOT after addition of H_2O_2 (100 μM) measured from the slope of the absorption.

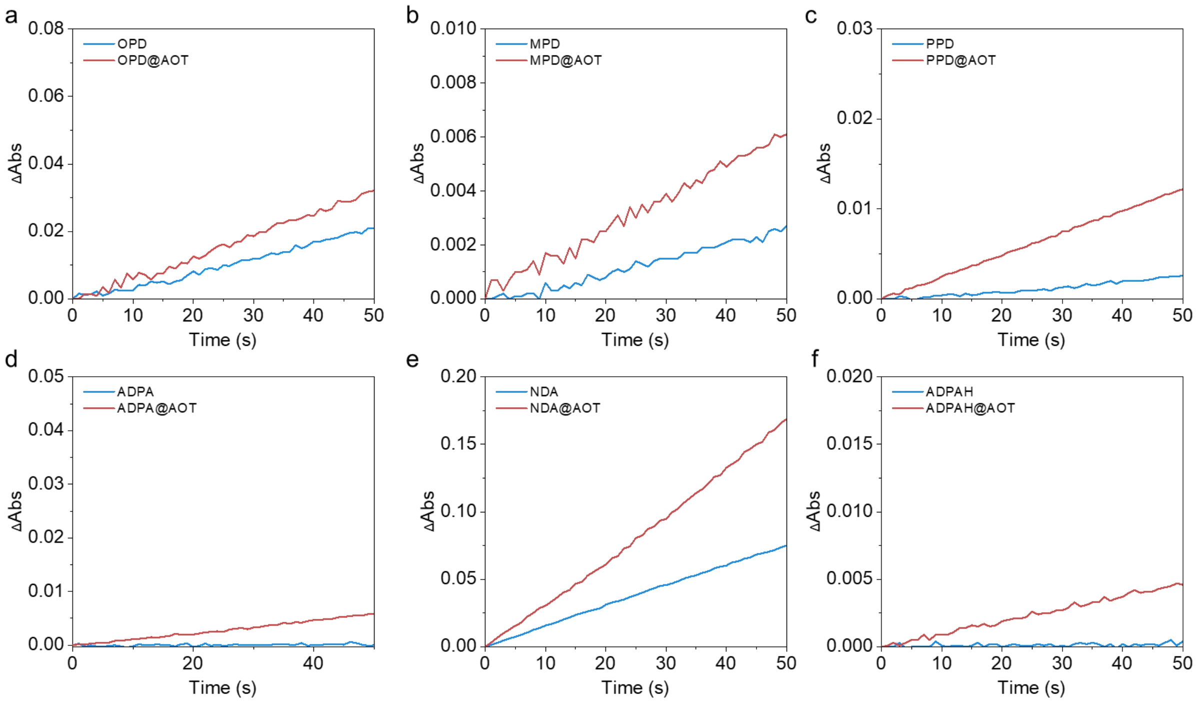

Supplementary Figure 5. Reaction rates. Reaction rates of **a** OPD and OPD@AOT, **b** MPD and MPD@AOT, **c** PPD and PPD@AOT, **d** ADPA and ADPA@AOT, **e** NDA and NDA@AOT, and **f** ADPAH and ADPAH@AOT after addition of H_2O_2 (100 μM) and fresh cell supernatant (10%) measured from the slope of the absorption.

	Initial velocity ratio[a]
$V_{OPD@AOT}/V_{OPD}$	1.3 ± 0.3
$V_{MPD@AOT}/V_{MPD}$	1.3 ± 0.1
$V_{PPD@AOT}/V_{PPD}$	2.4 ± 0.2
$V_{ADPA@AOT}/V_{ADPA}$	11.2 ± 0.5
$V_{NDA@AOT}/V_{NDA}$	1.8 ± 0.1
$V_{ADPAH@AOT}/V_{ADPAH}$	6.4 ± 0.4

[a]Initial velocity ratio: absorbance slope with AOT vesicles/absorbance slope without AOT vesicles × 100%

Supplementary Figure 6. Initial velocity ratios of the enzymatic reactions with or without AOT vesicles.

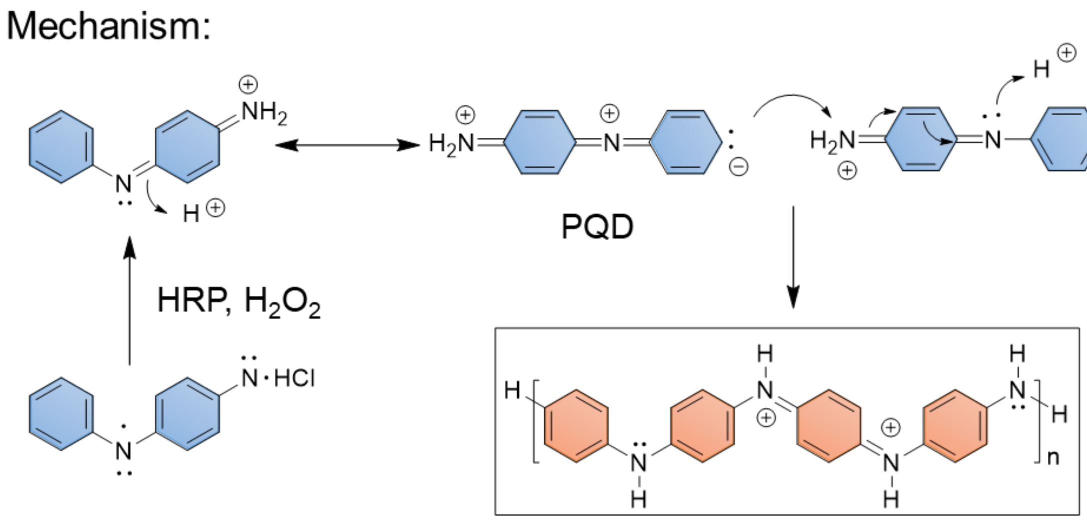

Supplementary Figure 7. Proposed mechanism for the polymerization of ADPAH.

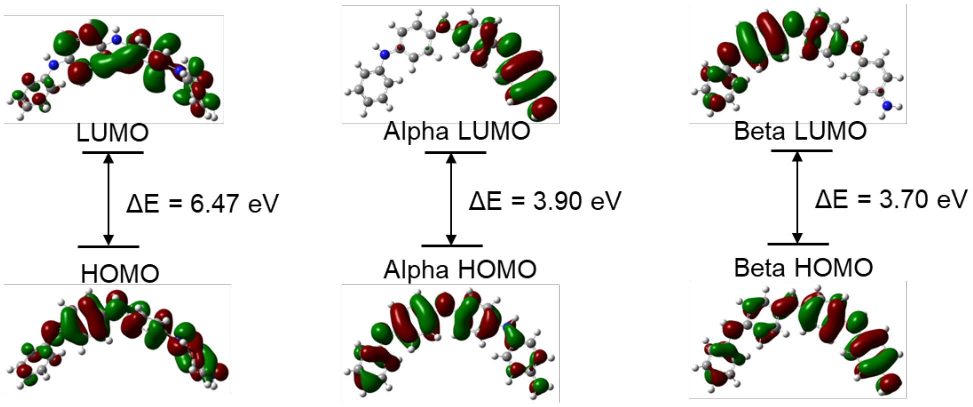

Supplementary Figure 8. LUMO−HOMO distribution of dimeric ADPAH and its radical structure.

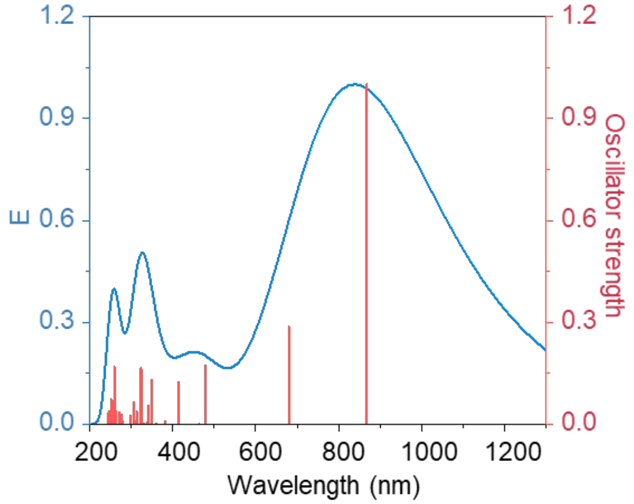

Supplementary Figure 9. Calculated absorption bands of proposed dimeric PADPAH.

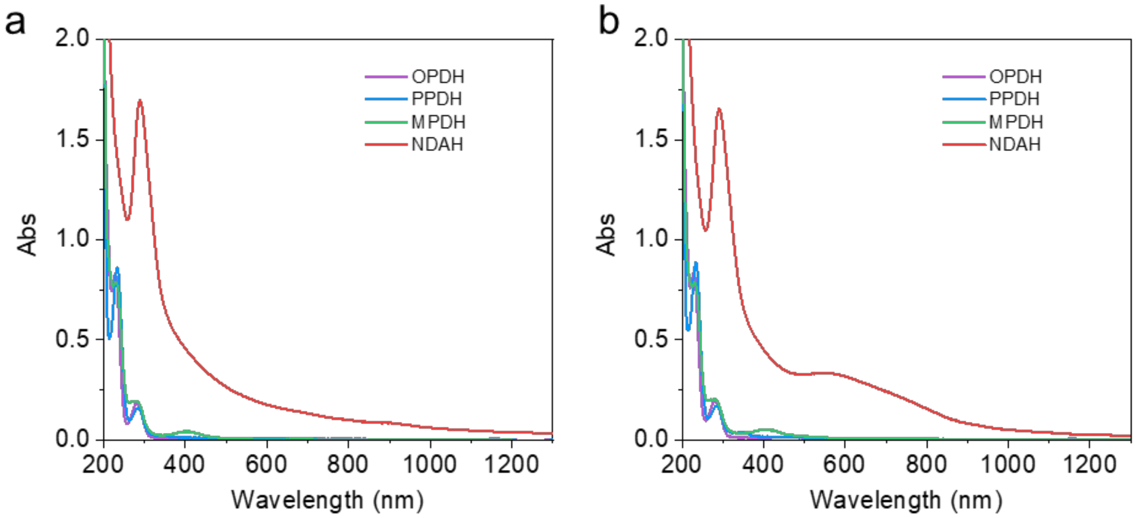

Supplementary Figure 10. Absorption spectra. Absorption spectra of OPDH, PPDH, MPDH, and NDAH **a** before and **b** after response to H_2O_2 (100 μM).

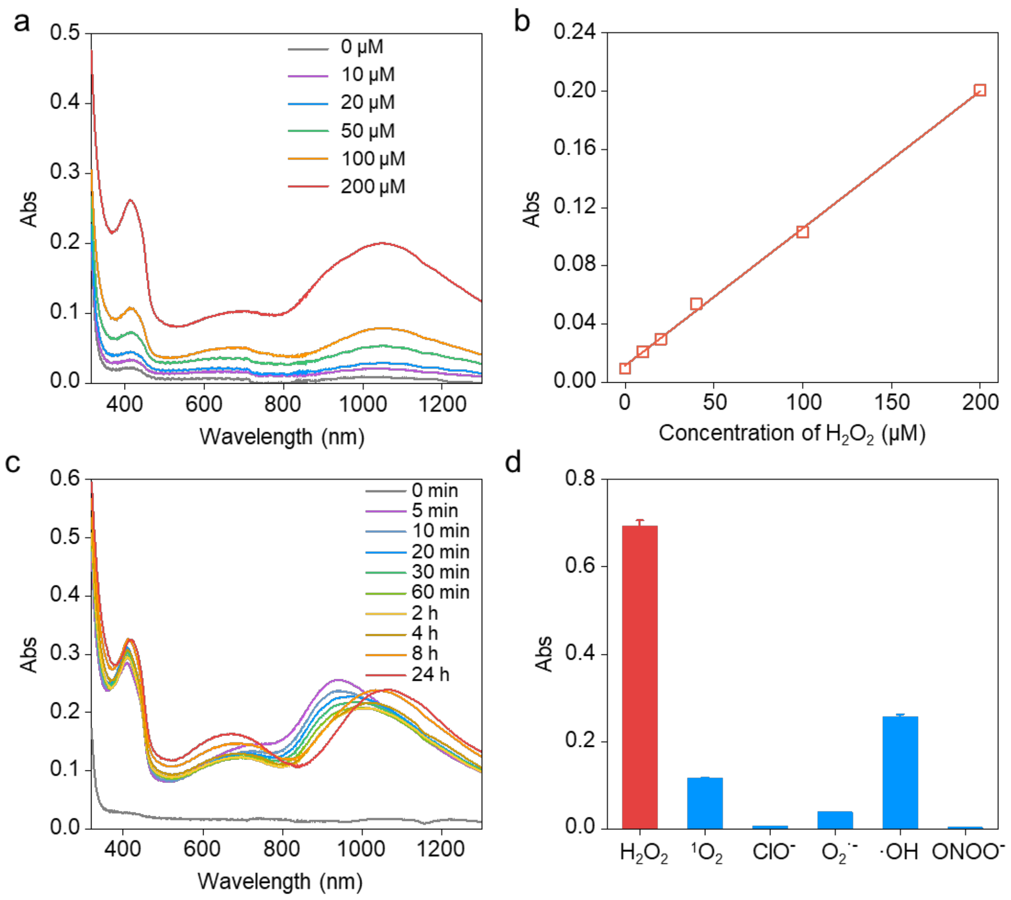

Supplementary Figure 11. Absorption spectra. a Absorbance spectra and **b** absorbance at 1064 nm of ADPAH@AOT dispersed in various concentrations of H_2O_2 (0, 10, 20, 50, 100, and 200 μM). **c** Absorbance spectra of ADPAH@AOT after response to H_2O_2 (100 μM) at different times. **d** Absorbance of ADPAH@AOT at 1064 nm after incubation with various types of ROS (100 μM) for 30 min. From the UV/vis−NIR spectrum, the oxidation polymerization of ADPAH was markedly improved with increasing H_2O_2 concentration in the range from 10 μM to 200 μM (Supplementary Fig. 11a). Additionally, the absorbance of ADPAH@AOT solutions at 1064 nm increased in proportion to the concentrations of added H_2O_2 (Supplementary Fig. 11b), showing the feasibility of quantification. With the increase of incubation time, the maximum absorption wavelength was gradually red-shifted, and the maximum absorption wavelength reached ~1070 nm after 24 h incubation (Supplementary Fig. 11c). The H_2O_2 specificity of this reaction was evaluated by incubating ADPAH@AOT with different types of ROS, including H_2O_2, singlet oxygen ($^1O_2$), hypochlorite (−OCl), superoxide ($O_2^{\cdot -}$), hydroxyl radical (·OH), and peroxynitrite anion ($ONOO^-$) (Supplementary Fig. 11d).

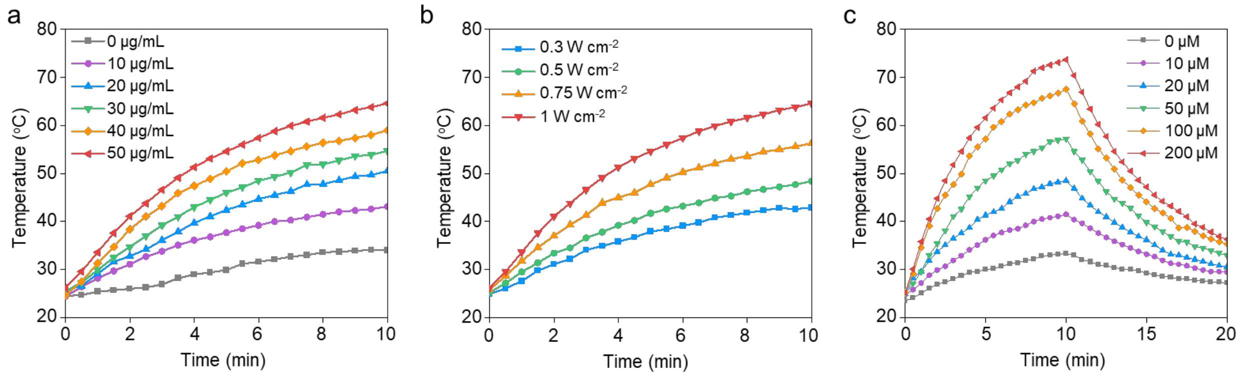

Supplementary Figure 12. Photothermal heating curves. a Photothermal heating curves of ADPAH@AOT with various concentrations of ADPAH (0, 10, 20, 30, 40, and 50 μM) responding to 1064 nm laser irradiation (1 W cm^{-2}); **b** Photothermal heating curves of ADPAH@AOT (50 μg mL^{-1}) under 1064 nm laser irradiation of different power densities (0.3, 0.5, 0.75 and 1 W cm^{-2}); **c** Photothermal heating curves of ADPAH@AOT with various concentration of H_2O_2 (0, 10, 20, 50, 100, and 200 μM) responding to 1064 nm laser irradiation (1 W cm^{-2}).

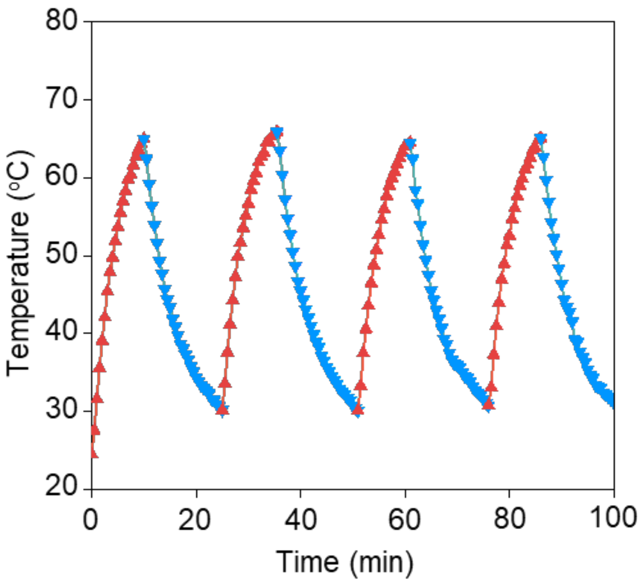

Supplementary Figure 13. Photothermal heating and natural cooling cycles of ADPAH@AOT in the presence of H_2O_2 (100 μM) under 1064 nm photoirradiation (1 W cm^{-2}).

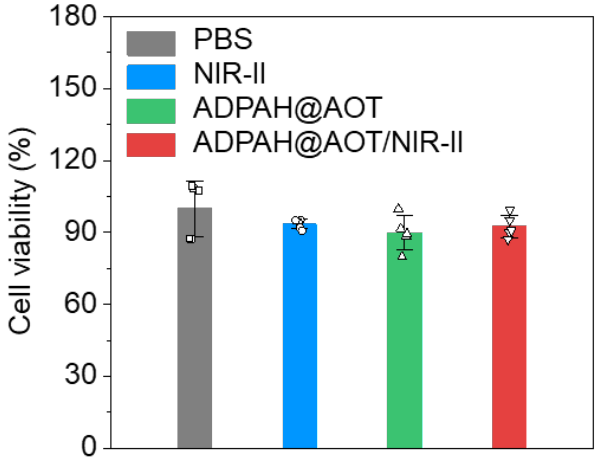

Supplementary Figure 14. Relative cell viabilities of NIH-3T3 cells after treatment with ADPAH@AOT with or without 1064 nm irradiation for 5 min (1 W cm^{-2}, n = 5).

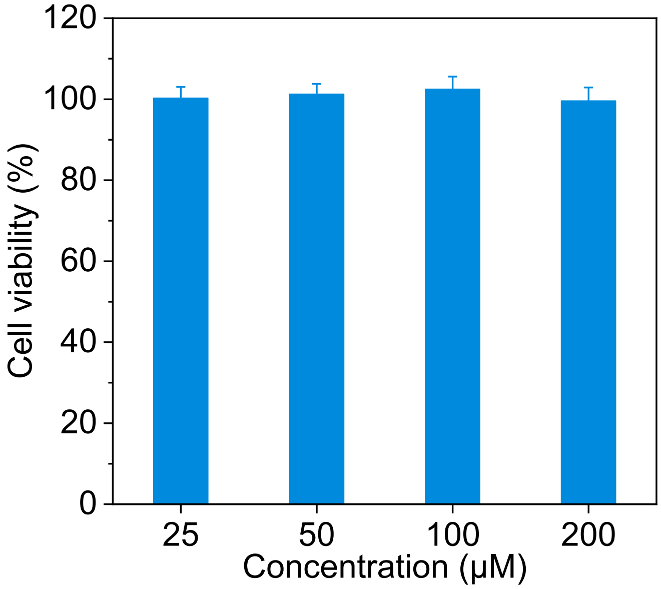

Supplementary Figure 15. Cell viability of 4T1 cells with different concentrations of H_2O_2 (n = 5).

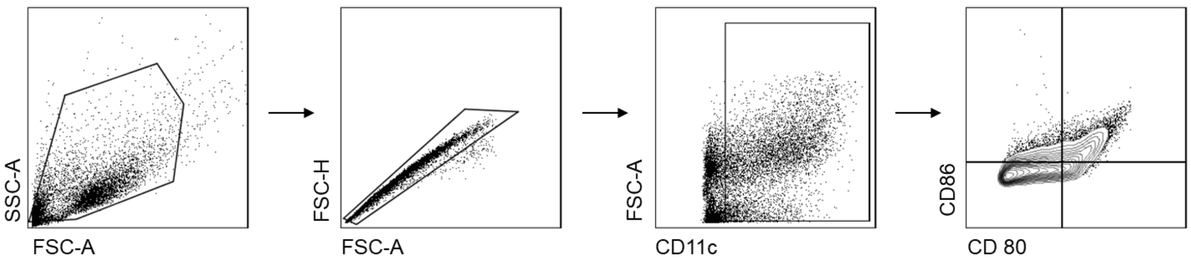

Supplementary Figure 16. Gating strategy to analyze matured DCs ($CD80^+$ and $CD86^+$) derived from bone marrow.

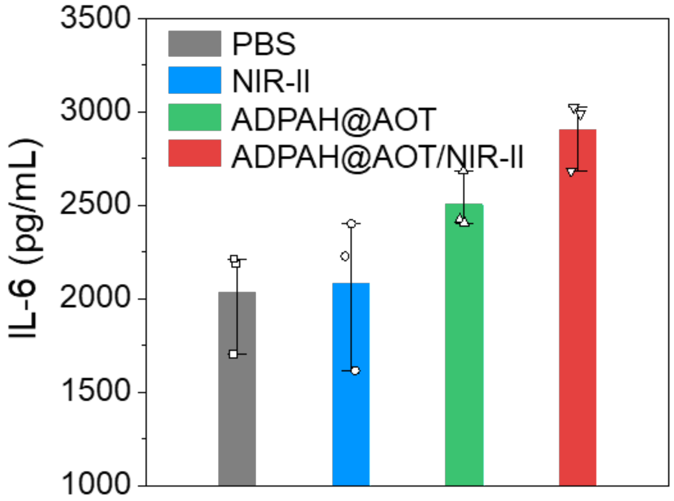

Supplementary Figure 17. Interleukin (IL)-6 content in DC suspensions from the transwell system (n = 3).

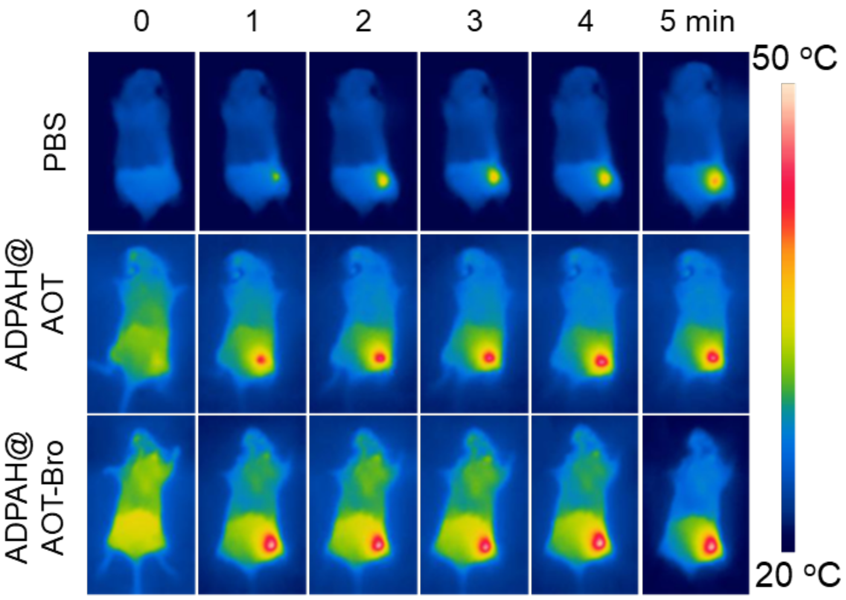

Supplementary Figure 18. Thermal images of 4T1-tumor-bearing mice under 1064 nm irradiation (1 W cm^{-2}) at 4 h post intertumoral injection of PBS, ADPAH@AOT, and ADPAH@AOT-Bro (n = 3).

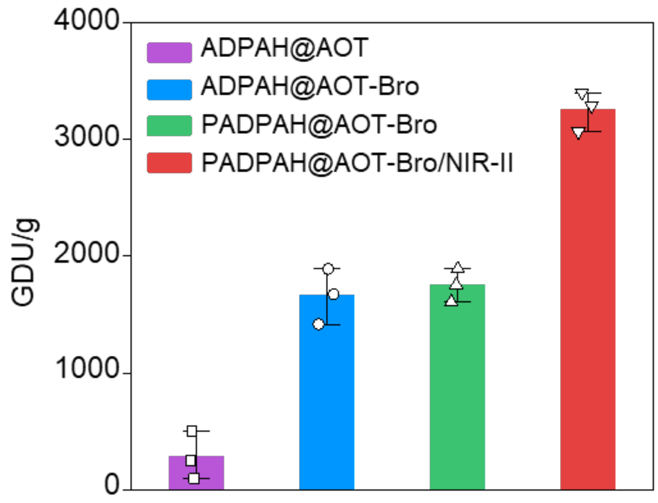

Supplementary Figure 19. Gelatin digestion activity (in GDU) of ADPAH@AOT with different treatments (n = 3).

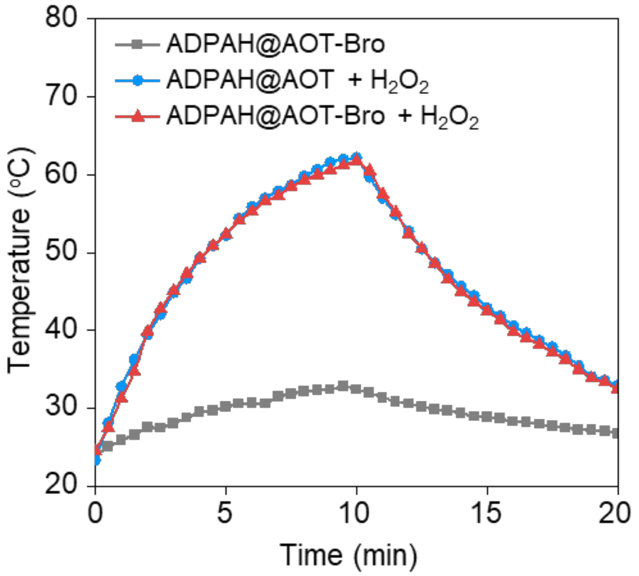

Supplementary Figure 20. Photothermal heating curves of ADPAH@AOT and ADPAH@AOT-Bro with responding to 1064 nm laser irradiation (1 W cm^{-2}).

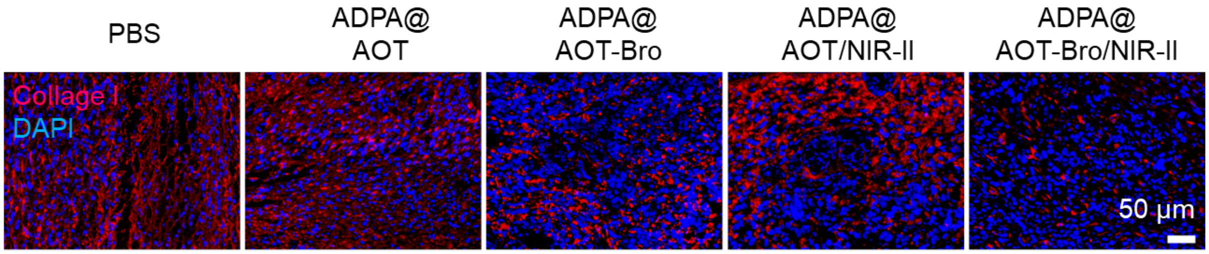

Supplementary Figure 21. Immunofluorescence staining of collage I from the primary tumor sections (n = 3).

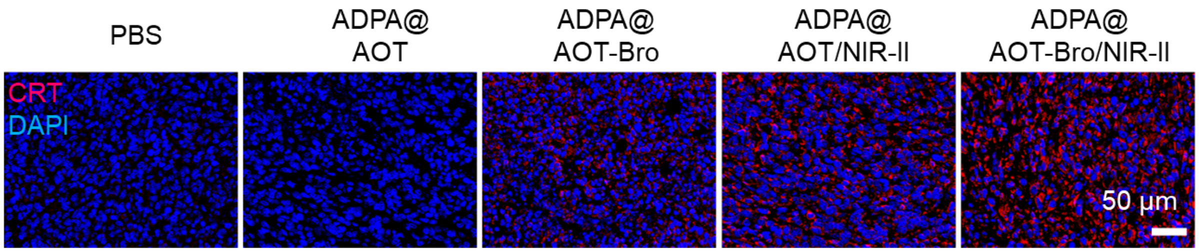

Supplementary Figure 22. Immunofluorescence staining of CRT from the primary tumor sections (n = 3).

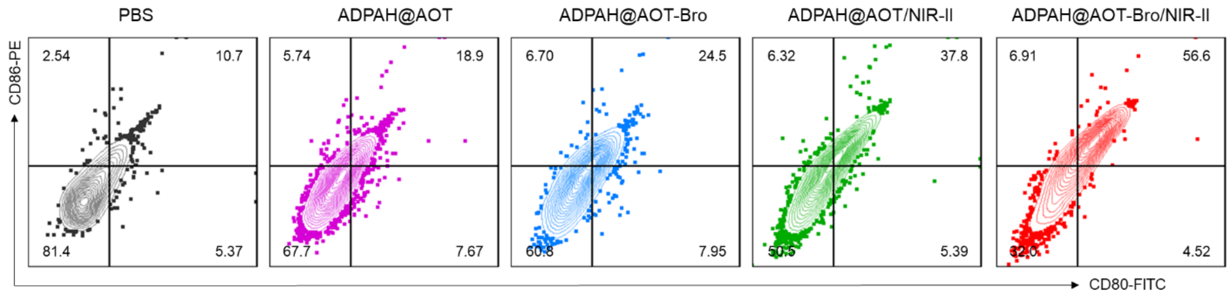

Supplementary Figure 23. Flow cytometric examination of DC maturation rates in the tumor-draining lymph nodes (gated on $CD11c^+$) from mice after different treatments indicated (n = 3).

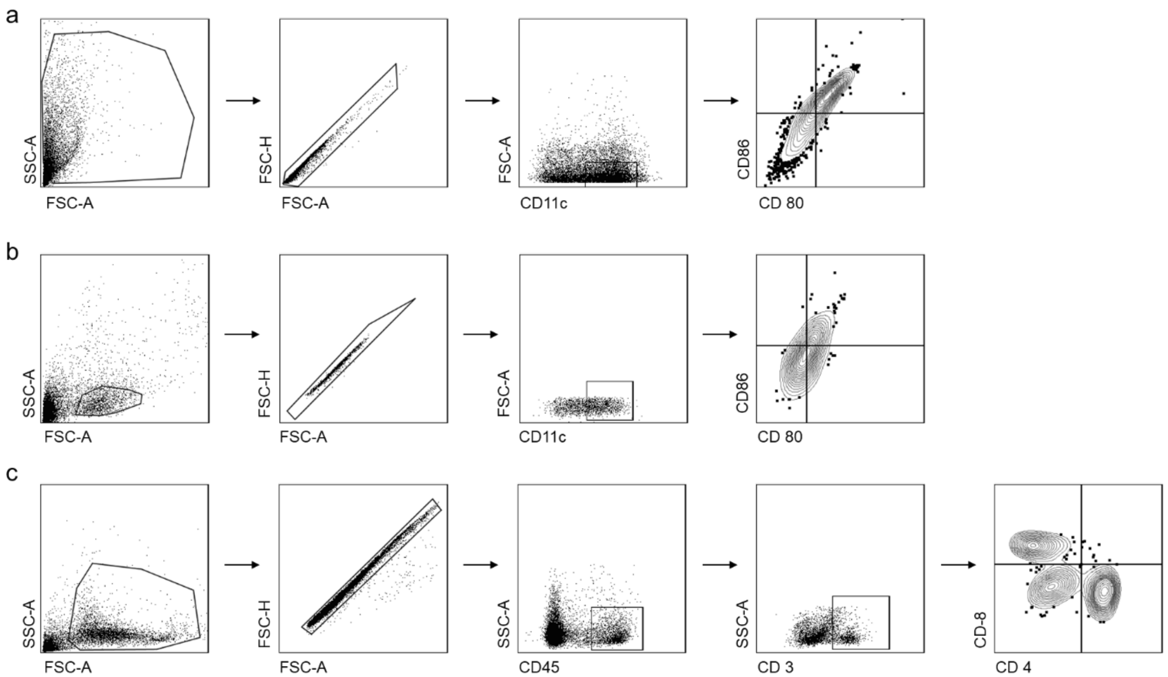

Supplementary Figure 24. Gating strategies used for flow cytometry analysis of immune cells in vivo. a Gating strategy to analyze matured DCs ($CD80^+$ and $CD86^+$) from tumor-draining lymph nodes (gated on $CD11c^+$) from mice after different treatments. **b** Gating strategy to analyze matured DCs ($CD80^+$ and $CD86^+$) from spleen (gated on $CD11c^+$) from mice after different treatments. **c** Gating strategy to analyze splenic T lymphocytes ($CD3^+CD8^+$ and $CD3^+CD4^+$) as a percentage of $CD3^+$ lymphocytes from spleens in 4T1 tumor-bearing mice after each treatment.

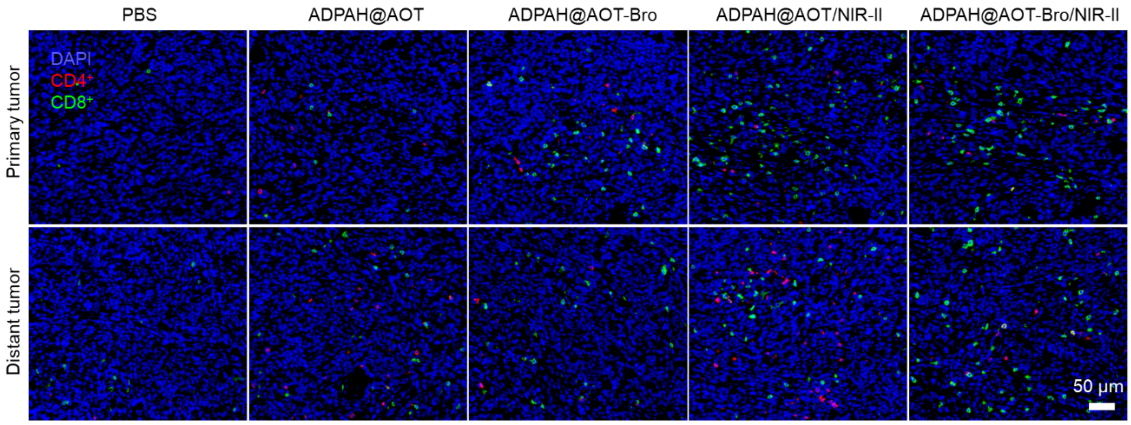

Supplementary Figure 25. Immunofluorescence staining of $CD4^{+}$ (red) and $CD8^{+}$ (green) in the primary and distant tumor tissues after various treatments (n = 3).

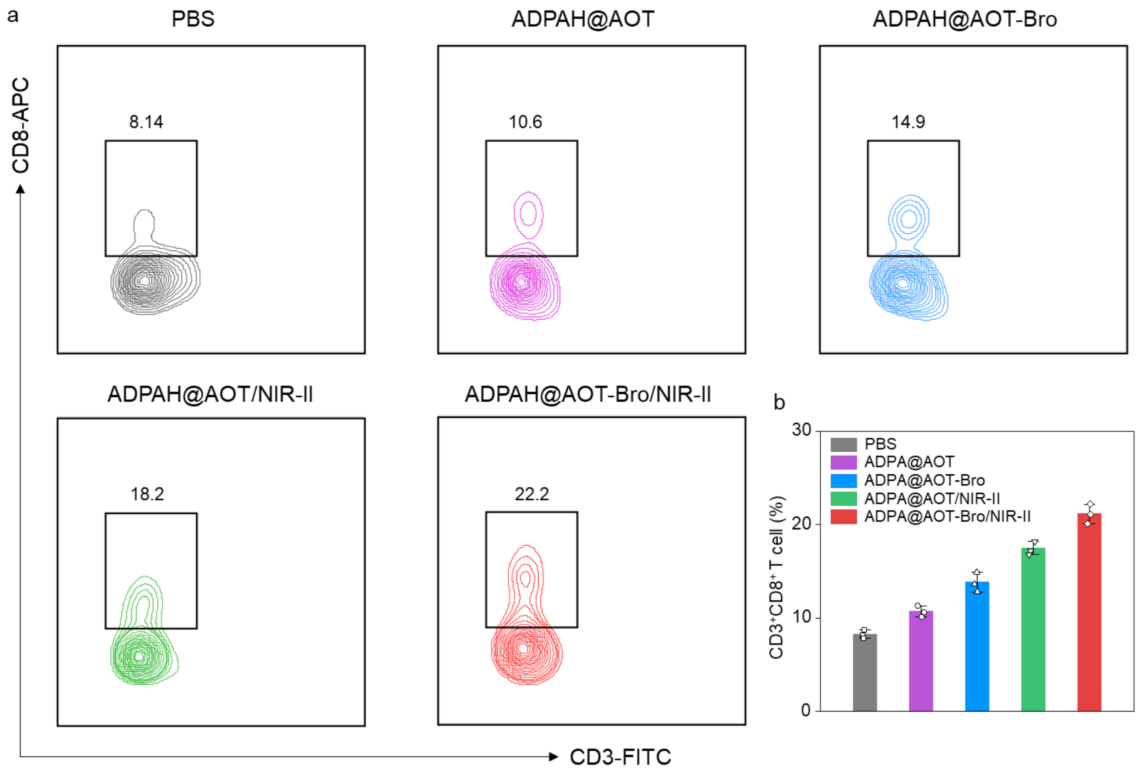

Supplementary Figure 26. Flow cytometry data. a Representative flow cytometry plots of cytotoxic T cells in primary tumors from mice at day 7 after photoirradiation; **b** Quantification of cytotoxic T cells in **a** (n = 3).

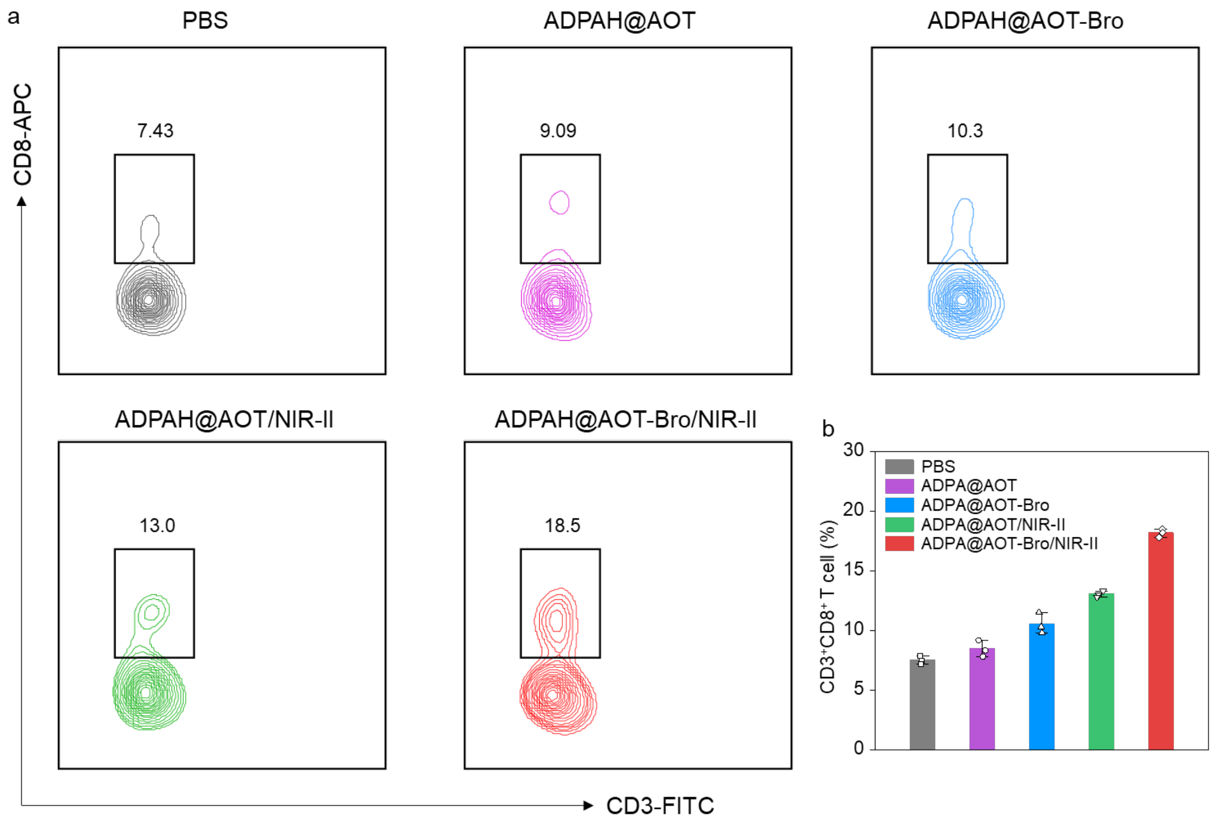

Supplementary Figure 27. Flow cytometry data. a Representative flow cytometry plots of cytotoxic T cells in distant tumors from mice at day 7 after photoirradiation; **b** Quantification of cytotoxic T cells in **a** (n = 3).

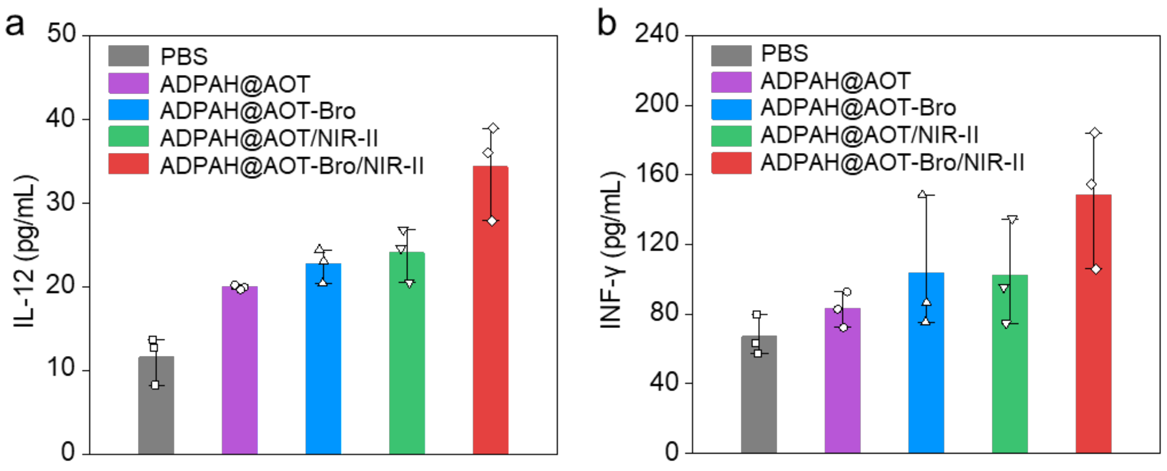

Supplementary Figure 28. Enzyme-linked immunosorbent assay. Enzyme-linked immunosorbent assay (ELISA) analysis of cytokines, **a** IL-12 and **b** interferon (IFN)-γ (n = 3).

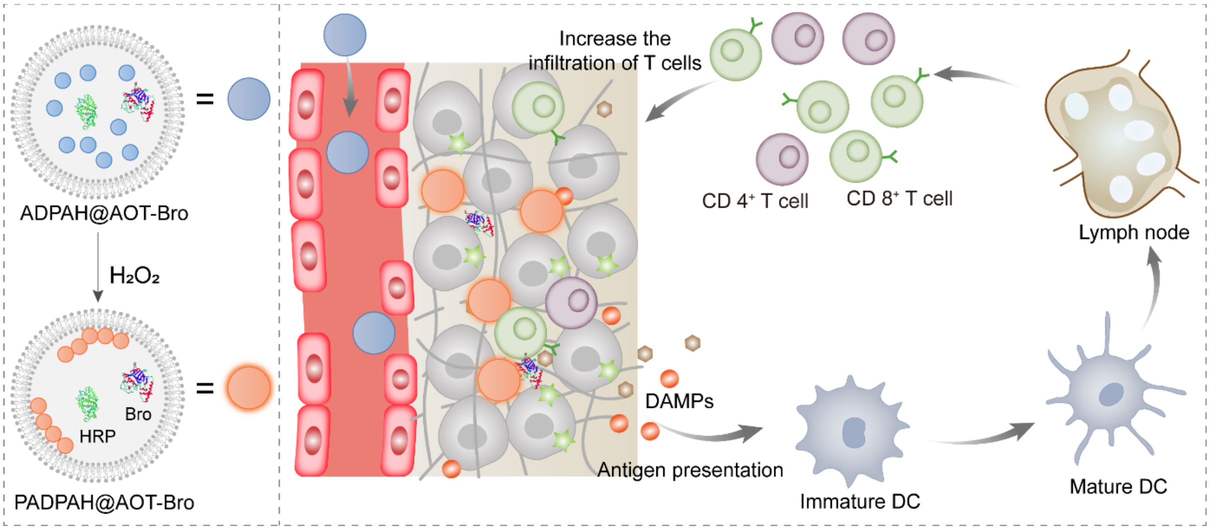

Supplementary Figure 29. Proposed pathway of the ADPAH@AOT-Bro mediated polymerization in living system and their role in NIR-II photothermal immunotherapy.

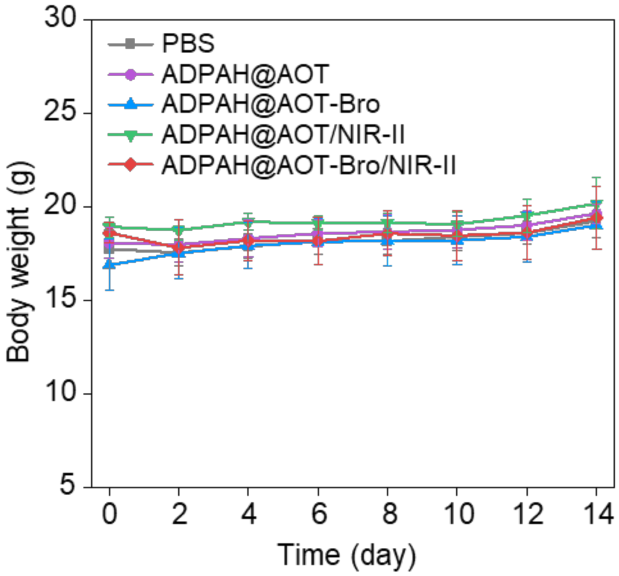

Supplementary Figure 30. Body weight changes of the mice under different treatments (n = 5).

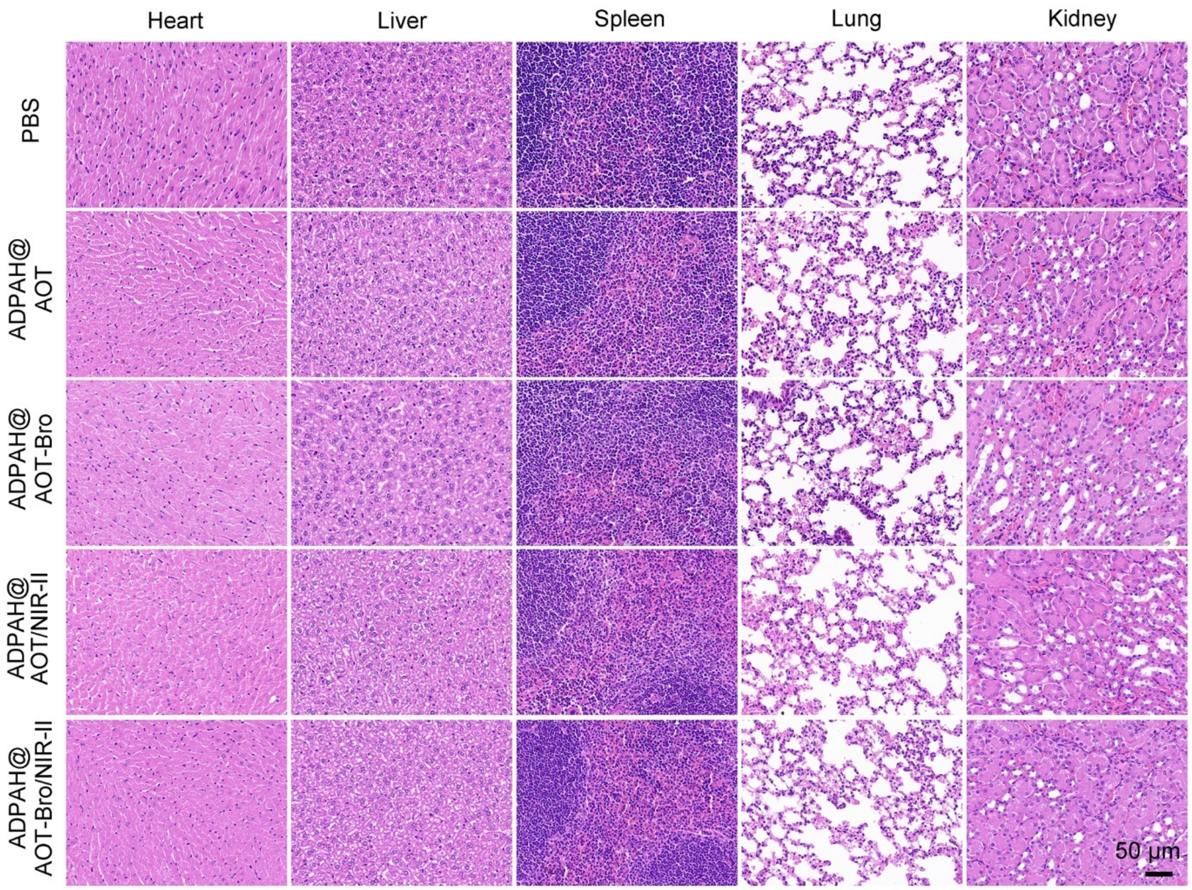

Supplementary Figure 31. Main organs (including heart, liver, spleen, lung, and kidney) examined through H&E staining after different treatments.
