# Supplementary material for: Nanocompartment-confined polymerization in living systems
Source: Nat Commun. 2023 Aug 26;14:5229. doi: 10.1038/s41467-023-40935-1 (PMC10460442; doi:10.1038/s41467-023-40935-1)
Supplement: Supplementary file 2 — Reporting Summary [file 41467_2023_40935_MOESM2_ESM.pdf]

nature portfolio

Corresponding author(s): Yanli Zhao; Wei Zhu; Peiyuan Yu

Last updated by author(s): Aug 5, 2023

Reporting Summary

Nature Portfolio wishes to improve the reproducibility of the work that we publish. This form provides structure for consistency and transparency in reporting. For further information on Nature Portfolio policies, see our Editorial Policies and the Editorial Policy Checklist.

Statistics

For all statistical analyses, confirm that the following items are present in the figure legend, table legend, main text, or Methods section.

n/a	Confirmed	
☐	☒	The exact sample size (*n*) for each experimental group/condition, given as a discrete number and unit of measurement
☐	☒	A statement on whether measurements were taken from distinct samples or whether the same sample was measured repeatedly
☐	☒	The statistical test(s) used AND whether they are one- or two-sided<br>*Only common tests should be described solely by name; describe more complex techniques in the Methods section.*
☐	☒	A description of all covariates tested
☐	☒	A description of any assumptions or corrections, such as tests of normality and adjustment for multiple comparisons
☐	☒	A full description of the statistical parameters including central tendency (e.g. means) or other basic estimates (e.g. regression coefficient) AND variation (e.g. standard deviation) or associated estimates of uncertainty (e.g. confidence intervals)
☐	☒	For null hypothesis testing, the test statistic (e.g. *F*, *t*, *r*) with confidence intervals, effect sizes, degrees of freedom and *P* value noted<br>*Give P values as exact values whenever suitable.*
☒	☐	For Bayesian analysis, information on the choice of priors and Markov chain Monte Carlo settings
☒	☐	For hierarchical and complex designs, identification of the appropriate level for tests and full reporting of outcomes
☒	☐	Estimates of effect sizes (e.g. Cohen's *d*, Pearson's *r*), indicating how they were calculated

Our web collection on statistics for biologists contains articles on many of the points above.

Software and code

Policy information about availability of computer code

Data collection	Transmission electron microscopy (TEM) images were collected by a JEOL JEM 2100plus microscopy. The fluorescence spectrum was obtained on an RF5301 PC fluorescence spectrometer. Ultraviolet/visible/near–infrared absorption spectra (UV–vis/NIR) absorption spectra were measured on a Shimadzu UV-3600 spectrometer. Confocal laser scanning microscopy images were acquired by ZEISS LSM 800 microscopy. Flow cytometry was performed with Fortessa X20 (BD Biosciences). The photoacoustic signal was measured by LOIS-3D (TomoWave Laboratories, USA).
Data analysis	Confocal fluorescence microscopy imaging data were analyzed using ZEN 2.3 blue edition (Carl Zeiss).<br>Flow cytometry data were performed using FlowJo software package (Flowjo V10).<br>Statistical analysis was calculated using GraphPad Prism V 9.5.0

For manuscripts utilizing custom algorithms or software that are central to the research but not yet described in published literature, software must be made available to editors and reviewers. We strongly encourage code deposition in a community repository (e.g. GitHub). See the Nature Portfolio guidelines for submitting code & software for further information.

Data

Policy information about availability of data

All manuscripts must include a data availability statement. This statement should provide the following information, where applicable:

- Accession codes, unique identifiers, or web links for publicly available datasets
- A description of any restrictions on data availability
- For clinical datasets or third party data, please ensure that the statement adheres to our policy

Source data are provided with this paper. The authors declare that the remaining data supporting the findings of this study are available within the article, its Supplementary Information and Source Data file, and the full image dataset is available from the corresponding author upon request.

Human research participants

Policy information about studies involving human research participants and Sex and Gender in Research.

Reporting on sex and gender	N/A
Population characteristics	N/A
Recruitment	N/A
Ethics oversight	N/A

Note that full information on the approval of the study protocol must also be provided in the manuscript.

Field-specific reporting

Please select the one below that is the best fit for your research. If you are not sure, read the appropriate sections before making your selection.

[x] Life sciences [ ] Behavioural & social sciences [ ] Ecological, evolutionary & environmental sciences

For a reference copy of the document with all sections, see nature.com/documents/nr-reporting-summary-flat.pdf

Life sciences study design

All studies must disclose on these points even when the disclosure is negative.

Sample size	All animal experiments were reviewed and approved with the Guidelines for Care and Use of Laboratory Animals of the Institutional Animal Care and Use Committee of Nanyang Technological University (NTU-IACUC) with a protocol number of A19016. Sample sizes were chosen based on previous experience with the animal models or following convention of the methods. We used G*power analysis to calculate and ensure the sample sizes fulfill adequate power ($p > 0.8$). According to the experimental data and sample size (n), P value and effect size were calculated and the power was then calculated. If it is higher than 80%, demonstrating the sample size is adequate.
Data exclusions	No animal and/or data excluded.
Replication	Experiments were repeated at least three independent experiments with similar results. All experiments were reproduced to reliably support conclusions stated in the manuscript.
Randomization	Allocation was random.
Blinding	The technician carried out the data collection and analysis without knowing the group assignment for each sample.

Reporting for specific materials, systems and methods

We require information from authors about some types of materials, experimental systems and methods used in many studies. Here, indicate whether each material, system or method listed is relevant to your study. If you are not sure if a list item applies to your research, read the appropriate section before selecting a response.

Materials & experimental systems

n/a	Involved in the study
☐	☒ Antibodies
☐	☒ Eukaryotic cell lines
☒	☐ Palaeontology and archaeology
☐	☒ Animals and other organisms
☒	☐ Clinical data
☒	☐ Dual use research of concern

Methods

n/a	Involved in the study
☒	☐ ChIP-seq
☐	☒ Flow cytometry
☒	☐ MRI-based neuroimaging

Antibodies

Antibodies used	Anti-Calreticulin antibody (cat no. ab227444, dilution of 1:200), Anti-HMGB1 antibody (cat no. ab79823, dilution of 1:250), Alexa Fluor 488 conjugated goat anti-rabbit IgG H&L (cat no. ab150077, dilution of 1:1000), and Goat Anti-Rabbit IgG H&L (cat no. Alexa Fluor® 594) (cat no. ab150080, dilution of 1:1000) were purchased from Abcam Inc. (Cambridge, CA, USA). Purified anti-mouse CD16/32 (cat no. 156604, dilution of 1:200), APC anti-mouse CD11c (cat no. 117310, dilution of 1:80), FITC anti-mouse CD80 (cat no. 104706, dilution of 1:50), PE anti-mouse CD86 (cat no. 105008, dilution of 1:200), FITC anti-mouse CD3 (cat no. 100204, dilution of 1:50), APC anti-mouse CD8a (cat no. 100712, dilution of 1:80), PE anti-mouse CD4 (cat no. 130310, dilution of 1:80), and Alexa Fluor®700 anti-mouse CD45 (cat no. 103128, dilution of 1:200) were purchased from Biolegend. Secondary antibody CY3-goat anti-rabbit IgG (cat no. GB21303, dilution of 1:300) was purchased from Servicebio.
Validation	All antibodies were used in the study according to the profile of manufacturers. Antibody validation was validated by the supplier and confirmed in this study.<br>https://www.biolegend.com/en-us/quality/product-development<br>https://www.abcam.com/primary-antibodies/how-we-validate-our-antibodies

Eukaryotic cell lines

Policy information about cell lines and Sex and Gender in Research

Cell line source(s)	Mouse breast cell line (4T1) and NIH-3T3 fibroblasts were purchased from ATCC.
Authentication	The cells were not authenticated before use.
Mycoplasma contamination	The cell line was tested negative through PCR detection.
Commonly misidentified lines (See ICLAC register)	No commonly misidentified lines were used.

Animals and other research organisms

Policy information about studies involving animals; ARRIVE guidelines recommended for reporting animal research, and Sex and Gender in Research

Laboratory animals	Balb/c mice (female, 5-6 weeks) were purchased from InVivos Pte. Ltd. (Singapore). Mice were housed in a temperature-constant animal room (22°C) with reversed dark/light cycle (7:00 a.m. on and 7:00 p.m. off) and 40–70% humidity.
Wild animals	No wild animals were used in this study.
Reporting on sex	This study did not involve sex consideration. Female mice were used to establish animal models according to literatures in this field.
Field-collected samples	No field-collected samples.
Ethics oversight	All animal experiments were reviewed and approved with the Nanyang Technological University Institutional Animal Care and Use Committee (NTU-IACUC).

Note that full information on the approval of the study protocol must also be provided in the manuscript.

Flow Cytometry

Plots

Confirm that:

- [x] The axis labels state the marker and fluorochrome used (e.g. CD4-FITC).
- [x] The axis scales are clearly visible. Include numbers along axes only for bottom left plot of group (a 'group' is an analysis of identical markers).
- [x] All plots are contour plots with outliers or pseudocolor plots.
- [x] A numerical value for number of cells or percentage (with statistics) is provided.

Methodology

Sample preparation	Depending on the experiment, 4T1 cells were grown to 80-90% confluence, harvested, and seeded in a 6 well plate and allowed to adhere overnight. Cells were cultivated in RPMI 1640 supplementing with 10 % (v/v) FBS and 1 % (w/v) penicillin (100 U/mL)/streptomycin (100 µg/mL) with 5% CO_2 at 37 °C. For all flow cytometry assays, following treatment or post-treatment incubation, cells were washed with PBS, centrifuged (1500 rpm × 5 min, 4 °C), collected, resuspended in cell binding solution (300 µL) and analyzed by FCM.<br>After various administrations, tumors were collected and cut into small pieces and incubated with the solution (containing 1 mg/mL collagenase I and IV, and 0.2 mg/mL DNase I) for 2 h at 37°C, and then the mixture was filtered through a strainer to get single cell suspension. The obtained cells were stained with suitable antibodies at 4 °C for 30 min and then detected by FCM. Spleen and tumor-draining lymph nodes were pressed gently and filtered through a strainer to get a single-cell suspension. The obtained cells were stained with suitable antibodies at 4 °C for 30 min and then detected by FCM.
Instrument	Fortessa X20 (BD Biosciences).
Software	Data analysis was done on FlowJo software (v10, FlowJo).
Cell population abundance	Cells were run to achieve > 10,000 events in the gated cell population.
Gating strategy	Gating was performed based on identifying a distinct population in FSC vs SSC plots.

- [x] Tick this box to confirm that a figure exemplifying the gating strategy is provided in the Supplementary Information.
